# Cerebrospinal fluid proteomics in patients with Alzheimer’s disease reveals five molecular subtypes with distinct genetic risk profiles

**DOI:** 10.1038/s43587-023-00550-7

**Published:** 2024-01-09

**Authors:** Betty M. Tijms, Ellen M. Vromen, Olav Mjaavatten, Henne Holstege, Lianne M. Reus, Sven van der Lee, Kirsten E. J. Wesenhagen, Luigi Lorenzini, Lisa Vermunt, Vikram Venkatraghavan, Niccoló Tesi, Jori Tomassen, Anouk den Braber, Julie Goossens, Eugeen Vanmechelen, Frederik Barkhof, Yolande A. L. Pijnenburg, Wiesje M. van der Flier, Charlotte E. Teunissen, Frode S. Berven, Pieter Jelle Visser

**Affiliations:** 1 Alzheimer Center Amsterdam, Neurology, Vrije Universiteit Amsterdam, Amsterdam UMC location VUmc, Amsterdam, the Netherlands; 2 Amsterdam Neuroscience, Neurodegeneration, Amsterdam, the Netherlands; 3 Proteomics Unit at the University of Bergen, Department of Biomedicine, University of Bergen, Bergen, Norway; 4 Department of Clinical Genetics, Vrije Universiteit Amsterdam, Amsterdam UMC location VUmc, Amsterdam, the Netherlands; 5 Center for Neurobehavioral Genetics, Semel Institute for Neuroscience and Human Behavior, David Geffen School of Medicine, University of California, Los Angeles, Los Angeles, CA USA; 6 Genomics of Neurodegenerative Diseases and Aging, Human Genetics, Vrije Universiteit Amsterdam, Amsterdam UMC location VUmc, Amsterdam, the Netherlands; 7 Department of Radiology and Nuclear Medicine, Vrije Universiteit Amsterdam, Amsterdam UMC location VUmc, Amsterdam, the Netherlands; 8 Amsterdam Neuroscience, Neuroimaging, Amsterdam, the Netherlands; 9 Neurochemistry Laboratory, Department of Laboratory Medicine, Vrije Universiteit Amsterdam, Amsterdam UMC location VUmc, Amsterdam, the Netherlands; 10 Delft Bioinformatics Lab, Delft University of Technology, Delft, the Netherlands; 11 Department of Biological Psychology, Vrije Universiteit Amsterdam, Amsterdam, the Netherlands; 12 ADx NeuroSciences, Ghent, Belgium; 13 Queen Square Institute of Neurology and Centre for Medical Image Computing, University College London, London, UK; 14 Epidemiology & Data Science, Vrije Universiteit Amsterdam, Amsterdam UMC location VUmc, Amsterdam, the Netherlands; 15 Alzheimer Center Limburg, School for Mental Health and Neuroscience, Maastricht University, Maastricht, the Netherlands; 16 Department of Neurobiology, Care Sciences and Society, Division of Neurogeriatrics, Karolinska Institutet, Stockholm, Sweden

**Keywords:** Diagnostic markers, Alzheimer's disease, Ageing

## Abstract

Alzheimer’s disease (AD) is heterogenous at the molecular level. Understanding this heterogeneity is critical for AD drug development. Here we define AD molecular subtypes using mass spectrometry proteomics in cerebrospinal fluid, based on 1,058 proteins, with different levels in individuals with AD (*n* = 419) compared to controls (*n* = 187). These AD subtypes had alterations in protein levels that were associated with distinct molecular processes: subtype 1 was characterized by proteins related to neuronal hyperplasticity; subtype 2 by innate immune activation; subtype 3 by RNA dysregulation; subtype 4 by choroid plexus dysfunction; and subtype 5 by blood–brain barrier impairment. Each subtype was related to specific AD genetic risk variants, for example, subtype 1 was enriched with *TREM2* R47H. Subtypes also differed in clinical outcomes, survival times and anatomical patterns of brain atrophy. These results indicate molecular heterogeneity in AD and highlight the need for personalized medicine.

## Abstract (web summary)

Tijms et al. identify five molecular Alzheimer’s disease subtypes typified by hyperplasticity, impaired immune activation, RNA metabolism, and choroid plexus or blood–brain barrier function. Subtypes may need tailored cures.

## Main

Alzheimer’s disease (AD) is the leading cause of dementia, affecting about 44 million people worldwide^1^. AD is histopathologically defined by amyloid plaques and hyperphosphorylated tau tangles in the brain, but its underlying pathophysiology is largely unclear. Genetic, brain tissue proteomics and gene expression studies indicated that many different pathophysiological processes are associated with amyloid and tau pathology, including but not limited to synaptic plasticity, the innate immune system, neuroinflammation, lipid metabolism, RNA metabolism, the matrisome and vascular function^2–8^. Differences between patients regarding the underlying mechanisms, together with other factors, may have contributed to limited or lack of clinical effects observed in previous AD trials^9–11^. For example, we previously found abnormally high cerebrospinal fluid (CSF) BACE1 levels in a specific AD subtype^8,12^, suggesting that BACE inhibition may be effective in a subgroup only, provided that other factors are optimized. This highlights the need for personalized treatments and for in vivo tools to define such molecular subtypes.

CSF is the most accessible biofluid to study the molecular complexity of neurodegenerative diseases during life. It is in close contact with the brain and protein concentrations in the CSF reflect the brain’s ongoing (patho)physiological processes. We previously discovered and replicated three distinct molecular AD subtypes by investigating the 707 and 204 proteins in the CSF^8^. The proteins involved in these subtypes represent distinct biological processes, such as neuronal plasticity, innate immune activation and blood–brain barrier dysfunction^8^. Subtype-specific molecular alterations were already present at a very early stage of AD, when cognition was still intact and neuronal damage still limited. Many of these molecular processes were also previously identified in AD postmortem tissue proteomics or gene expression studies^2,3,5,7^. This supports the value of CSF proteomics to detect AD pathophysiological processes in living patients^3^.

Proteomic techniques have greatly improved since and can detect thousands of proteins in the CSF, which provides an opportunity to dissect the molecular processes associated with AD subtypes in detail. In this study, we used these techniques and detected more than 3,000 proteins in the CSF in another independent cohort of 609 individuals to replicate and refine the existing subtypes, to test if the higher complexity allows us to uncover additional AD subtypes and to study the underlying genetic factors of these subtypes.

In our previous studies, we compared CSF AD subtypes on *APOE* e4 carriership (the strongest genetic risk factor for sporadic AD)^8,13^ and on AD polygenic risk scores (PRS). In the current study, we further extended the genetic analyses and compared subtypes on AD risk variants from a recent genome-wide association study (GWAS)^4^. Moreover, we enriched for the relatively rare *TREM2* R47H and R62H mutations because these are associated with a 2.3 and 1.4-fold increased risk for AD^4^. *TREM2* R47H and R62H impair microglial activation in AD^14^. Therefore, we hypothesized that *TREM2* carriers could be grouped together in a subtype with impaired microglial activation. A small number of patients (*n* = 6) carried an autosomal dominant mutation in *PSEN1* or *APP*; we performed an exploratory analysis to identify which subtypes these genetic variants were associated with.

This large-scale CSF proteomic study revealed five molecular AD subtypes. Three subtypes recapitulated our previously identified three subtypes (hyperplasticity, innate immune activation and blood–brain barrier dysfunction)^8^. We further identified two additional AD subtypes: one with RNA dysregulation and one with choroid plexus dysfunction. All subtypes were associated with distinct genetic risk profiles, providing further biological validation for AD subtypes. The proteomic signatures associated with AD subtypes were present already at the preclinical stage and largely remained stable with increasing disease severity. Subtypes differed in the amount and pattern of cortical atrophy, cell type-specific expression of proteins, vascular damage and clinical outcomes. These results highlight the importance of neuronal plasticity, microglial impairment, innate immune activation, RNA processing, choroid plexus and blood–brain barrier dysfunction in AD pathogenesis, and provide a comprehensive resource that informs on the proteins and pathways that are dysregulated in patients with a specific AD subtype.

## Results

We analyzed CSF samples from 609 individuals that were selected from the Alzheimer Center Amsterdam related studies^15–18^ (for clinical details, see Methods and Supplementary Table 1). Of this sample, 419 individuals had AD as defined by an abnormal amyloid biomarker and included all clinical stages (that is, 107 with normal cognition, 103 with mild cognitive impairment (MCI) and 209 with dementia). The 187 controls were required to have normal cognition and normal amyloid and tau biomarkers. CSF proteins from each sample were enzymatically digested and the peptides were labeled with tandem mass tags (TMTs), fractionated and analyzed by liquid chromatography–tandem mass spectrometry (LC–MS/MS) (Methods). A total of 3,863 proteins was identified, of which 1,309 proteins (defined by 28,408 peptides) were observed across all individuals. We then tested which proteins had different levels in individuals with AD compared to controls, and repeated those analyses stratified on tau levels or disease stage because protein levels can change in a nonlinear way with these variables^12,19^. This led to the selection of 1,058 AD-related proteins for cluster analyses (Supplementary Table 2; it also includes information at the peptide level). We then clustered individuals with AD on AD-related proteins with nonnegative matrix factorization^20^, which is a dual clustering approach (Supplementary Fig. 1). A particular strength of the algorithm is that individuals are per definition allocated to one subtype, which is useful for diagnosis or patient stratification for trials.

### Five AD subtypes that differ on clinical characteristics

**Fig. 1:**
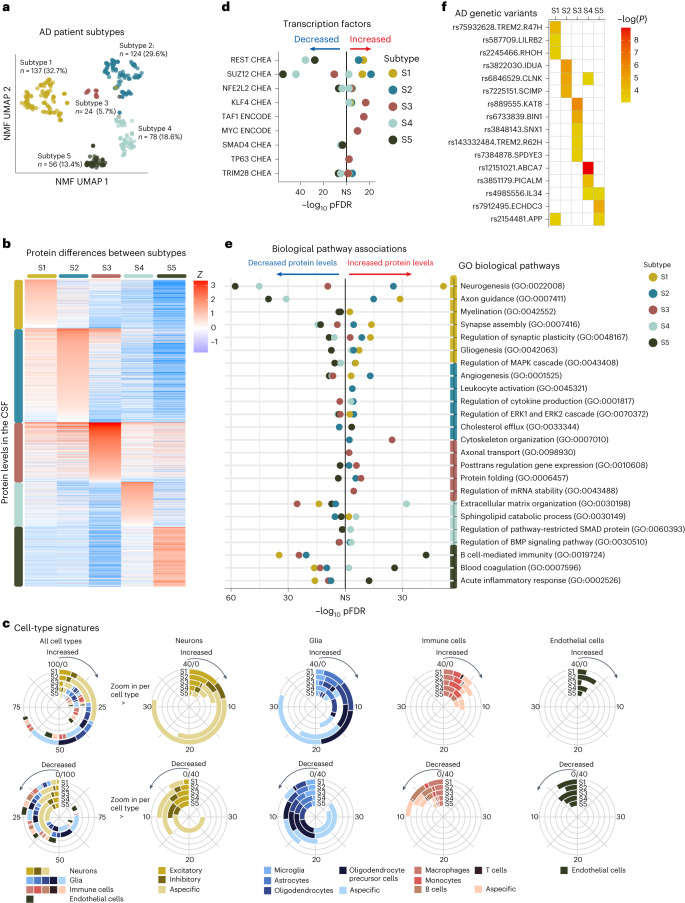
Biological description of AD subtypes. **a**, Patient subtypes projected to the uniform manifold approximation and projection (UMAP) space. **b**, CSF protein levels (rows) averaged across individuals within subtypes (columns). **c**, Cell-type-specificity signatures for proteins associated with the AD subtypes for proteins with increased (top row) and decreased (bottom row) level. The left circle diagram shows all cell types associated with a subtype combined. Proteins that could not be assigned to a specific cell type were not plotted (no colour to 100% in the left circle diagrams). The circle diagrams to the right zoom into the subcategories of specific cell types (neurons, glia, immune cells and endothelial cells). Cell-type specificity was determined according to the Human Protein Atlas. **d**, Top transcription factors associated with subtypes from the CHEA and ENCODE databases. **e**, Gene Ontology (GO) biological pathways associated with subtypes (see Supplementary Table 9 for all pathways). **f**, AD genetic risk factors associated with specific subtypes; white indicates not statistically significant. Differences between subtypes and controls were determined from linear regression models with estimated marginal means, providing a two-tailed test for group comparisons, uncorrected for multiple testing because this is a post hoc comparison. Supplementary Tables 4 and 9–11 list all the proteins, pathways, and transcription and genetic factors tested with the statistical metrics. NS, not statistically significant; S1, subtype 1 (hyperplasticity); S2, subtype 2 (innate immune activation); S3, subtype 3 (RNA dysregulation); S4, subtype 4 (choroid plexus dysfunction); S5, subtype 5 (blood–brain barrier dysfunction).

**Fig. 2:**
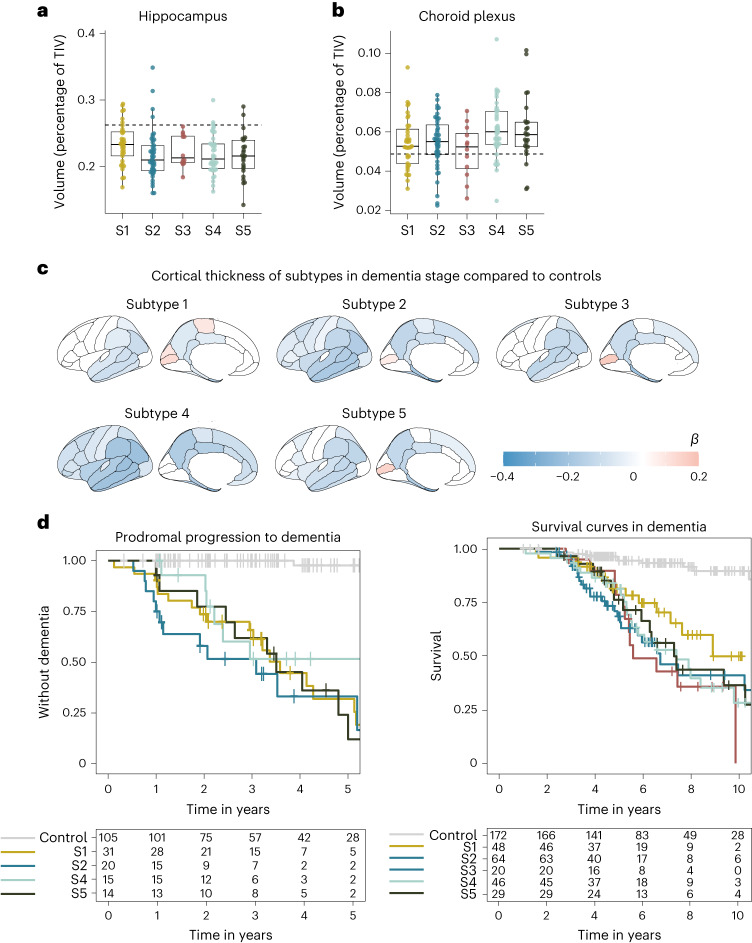
AD subtype comparisons on MRI and clinical outcomes. **a**, Median hippocampal volume as the percentage of total intracranial volume (TIV) compared to subtypes in the dementia stage. **b**, Choroid plexus volume as the percentage of TIV compared to subtypes in the dementia stage. **c**, Cortical atrophy associated with AD subtypes in the dementia stage compared to controls (*n* = 160). *β* indicates mean cortical thickness in mm, averaged over the right and left hemispheres and adjusted for age and sex. **d**, Clinical progression from MCI to dementia according to subtype (left; excluding subtype 3 due to *n* = 2) and time from dementia to death according to subtypes (right). All atrophy measures are based on individuals with dementia only. **a**,**b**, The boxplots depict the median in the center; the boundaries indicate the first and third quartiles, while the whiskers extend up and down to 1.5 times the interquartile range (limited to actual observed data points), and the points indicate individual person values (subtype 1, *n* = 37; subtype 2, *n* = 45; subtype 3, *n* = 12; subtype 4, *n* = 40; subtype 5, *n* = 25). See Supplementary Tables 6a,b and 8 for the detailed statistical metrics.

**Extended Data Fig. 1:**
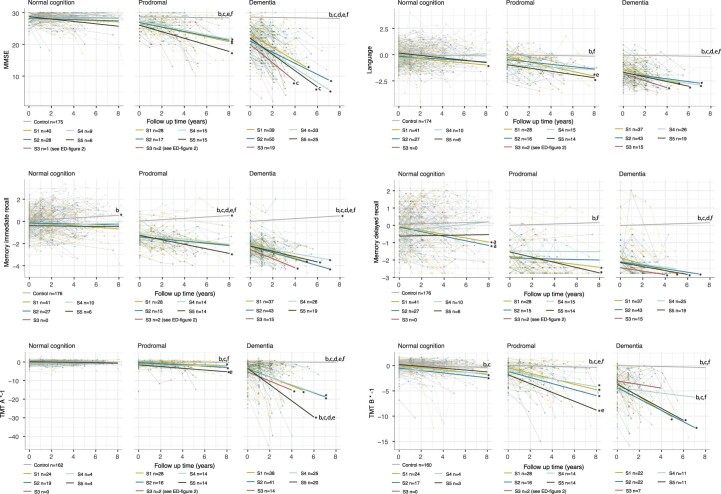
Trajectories of repeated cognitive test scores over time for AD subtypes. Analyses were stratified according to clinical stage. In all plots the grey line represents the trajectory of the control group (that is, individuals with intact cognition and normal CSF markers). * indicates that the slope differs from zero with p < 0.05. a indicates slope different from controls, b indicates slope different from subtype 1, c indicates slope different from subtype 2, d indicates slope different from subtype 3, e indicates slope different from subtype 4 and f indicates slope different from subtype 5. See Supplementary Table 7 for statistical metrics.

**Extended Data Fig. 2:**
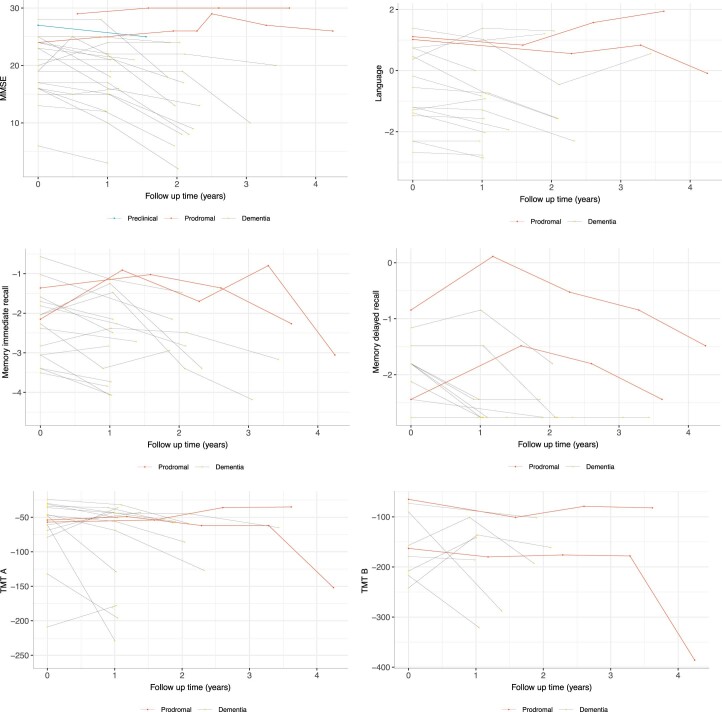
Trajectories of repeated cognitive scores for subtype 3 individuals without dementia. Grey lines indicate individuals with dementia and subtype 3. MMSE includes one individual with normal cognition (blue line), all other tests include two individuals with MCI (red lines). No statistics were performed for predementia individuals due to small sample size.

The patients’ proteomic profiles clustered into five subtypes (Fig. 1a and Supplementary Table 3 for the fit and stability statistics): subtypes 1, 2 and 5 recapitulated our previously detected subtypes with neuronal hyperplasticity (subtype 1), innate immune activation (subtype 2) and blood–brain barrier dysfunction (subtype 5); two additional subtypes emerged: one with RNA dysregulation (subtype 3) and one with choroid plexus dysfunction (subtype 4). We tested the robustness of the subtypes by clustering the weighted protein coexpression network again with the Louvain algorithm (Methods), which also resulted in five protein clusters that were similar to the NMF protein clusters (93.5% overlap of cluster-specific proteins (Supplementary Table 4, column X). The next sections discuss each subtype in detail according to molecular, genetic and clinical characteristics. We briefly summarize the subtype differences. Subgroups were compared to controls and each other, with estimated marginal means from linear models for continuous outcomes, which were two-tailed tests, and proportions were tested with chi-squared tests and repeated for each pairwise group combination (Table 1, please note these comparisons are uncorrected for multiple testing because this is a descriptive table). Compared to controls, subtypes 1, 2 and 3 had increased CSF t-tau and p-tau levels, while subtypes 4 and 5 had mostly normal tau levels (see Table 1 and Supplementary Table 5for additional analyses stratified according to cognitive state and adjusted for sex and age). Subtypes differed according to clinical stage, sex and age; all subsequent analyses took these characteristics into account. Compared to controls, subtypes differed in the rates of progression from MCI to dementia, with subtypes 2 and 5 having the highest risk, and subtype 4 the lowest (Fig. 2d), although differences between subtypes did not reach statistical significance (Supplementary Table 6a). Subtype 3 individuals with dementia had the shortest average survival time of 5.6 years, which was shorter than subtype 1 with the longest average survival time of 8.9 years (*P* = 0.04; Fig. 2 and Supplementary Table 6b), and steeper decline on Mini Mental State Examination (MMSE), language and memory tests (Extended Data Figs. 1 and 2 and Supplementary Table 7). These results suggests that different underlying molecular processes may explain a part of between-patient variability in decline. Analysis of magnetic resonance imaging (MRI) scans in individuals with dementia (*n* = 159) indicated that subtypes differed in the degree and anatomical location of cortical atrophy (Fig. 2 and Supplementary Table 8). All subtypes had a higher prevalence of the APOE e4 genotype than controls, and a higher AD PRS, supporting their underlying AD genetic risk architecture. However, subtypes had distinct AD genetic risk profiles (discussed in detail below).

**Table 1:** Comparison of subtypes according to clinical characteristics

Characteristic	Controls *n* = 187	Subtype 1 *n* = 137	Subtype 2 *n* = 124	Subtype 3 *n* = 24	Subtype 4 *n* = 78	Subtype 5 *n* = 56
Cognitive state, *n* (%)						
Normal cognition	187 (100)	51 (37)	32 (26)	1 (4)^a,b^	15 (19)^a^	8 (14)^a^
MCI	0	37 (27)	28 (23)	3 (12)	16 (21)	19 (34)
Dementia	0	49 (36)	64 (52)^a^	20 (83)^a,b^	47 (60)^a^	29 (52)^c^
Age	64.01 (11.83)	64.71 (6.82)	69.38 (8.35)^a,d^	64.46 (8.73)^b^	64.28 (8.09)^b^	66.16 (8.11)^b^
Men, *n* (%)	111 (59)	62 (45)^d^	61 (49)	10 (42)	51 (65)^a,b^	41 (73%)^a,b,c^
Years of education, mean (s.d.)	12.4 (3.2)	12.1 (3.4)	11.2 (3.2)^d^	11.7 (2.9)	11.9 (3.4)	11.8 (3.3)
≥1 *APOE* e4 allele, *n* (%)	51 (28)	88 (68)^d^	73 (62)^d^	15 (65)^d^	47 (64)^d^	40 (74%)^d^
AD PRS, mean (s.d.)	5.6 (0.37)	5.8 (0.41)^d^	5.8 (0.31)^d^	6.0 (0.41)^d,b^	5.8 (0.46)^d^	5.8 (0.34)^d^
CSF total tau, pg ml^−1^, mean (s.d.)	199 (88)	592 (340)^d^	765 (447)^a,d^	882 (367)^a,d^	301 (166)^a,b,c,d^	469 (297)^a,b,c,d,e^
CSF p-tau 181, *z*-score, mean (s.d.)	0 (0.99)	3.4 (2.5)^d^	5.1 (3.1)^a,d^	5.0 (2.5)^a,d^	0.6 (1.4)^a,b,c,d^	2.1 (2.3)^a,b,c,d,e^
CSF BACE1, pg ml^−1^, mean (s.d.)	1931.9 (643.49)	2203.8 (479.07)^d^	2478.49 (687.98)^a,d^	2185.33 (573.92)^b,d^	1391.36 (323.74)^a,b,c,d^	1819.55 (560.55)^a,b,c,e^
CSF Abeta40, pg ml^−1^, mean (s.d.)	7135.12 (2134.68)	7825.31 (1722.1)^d^	8519.82 (2264.92)^a,d^	6817.46 (1783.25)^a,b^	4610.78 (1286.96)^a,b,c,d^	5943.02 (1543.58)^a,b,c,d,e^
CSF NRGN, pg ml^−1^, mean (s.d.)	317.49 (148.07)	488.01 (178.95)^d^	634.44 (300.5)^a,d^	561 (175.88)^b,d^	244.09 (97.64)^a,b,c,d^	370.91 (166.83)^a,b,c,e^
CSF NEFL, pg ml^−1^, mean (s.d.)	360.1 (275.1)	447.01 (187.34)^d^	620.08 (341.41)^a,d^	630.12 (293.84)^a,d^	453.61 (292.84)^b,c,d^	594.16 (371.67)^a,d,e^
CSF VAMP2, pg ml^−1^, mean (s.d.)	162.17 (70.43)	196.32 (61.39)^d^	233.44 (79.91)^a,d^	188.6 (61.77)^b^	100.1 (39.8)^a,b,c,d^	141.94 (52.35)^a,b,c,d,e^
Microbleed count on MRI, mean (s.d.)	0.91 (2.55)	1.89 (9.80)	1.16 (3.74)	1.65 (3.18)	2.07 (8.02)	4.40 (17.94)^b,d^

^a^Differs from subtype 1 with *P* < 0.05.

^b^Differs from subtype 2 with *P* < 0.05.

^c^Differs from subtype 3 with *P* < 0.05.

^d^Differs from controls with *P* < 0.05.

^e^Differs from subtype 4 with *P* < 0.05.

We next examined the molecular processes associated with the AD subtypes. For each subtype, we compared the levels of 2,878 proteins against the control group (Fig. 1b and Supplementary Table 4). Proteins with different levels between a subtype and the control group were included in the enrichment analyses to study associated biological processes and transcription factors. To aid comparability with the gene expression literature, we report gene names for proteins (see Supplementary Table 4 for the UniProt codes). Stratification according to clinical stage resulted in similar differences to controls (correlations of effects ranging between 0.85 and 0.98; Extended Data Fig. 4), further supporting that AD subtypes reflect specific disease traits^8,13^.

Below, we highlight subtype-specific associations with biological processes, cell types, AD genetic risk variants and atrophy patterns (see Supplementary Tables 4–11 for detailed results).

### Subtype 1 hyperplasticity

Subtype 1 individuals (*n* = 137, 32.7%) had 827 proteins with increased CSF levels and 408 proteins with decreased levels compared to controls. Of all the subtypes, subtype 1 had the highest proportion of proteins specific for neurons, astrocytes, oligodendrocytes and oligodendrocyte precursor cells (Fig. 1c). Proteins with increased levels were associated with neuronal plasticity processes, including synapse assembly, axon guidance, neurogenesis and gliogenesis (Fig. 1e; see Supplementary Table 9 for all biological processes enriched). In addition, this neuronal hyperplasticity subtype had high BACE1, amyloid-β_1–40_ and tau CSF levels (Table 1), as we previously observed in the hyperplasticity subtype^8^. While high tau levels were previously thought to reflect neuronal loss due to tangle formation, more studies are indicating that this may also reflect other processes^21,22^. For example, neurons with increased activity secrete more amyloid and tau^23–27^; such hyperactive neurons have been observed near plaques^28^. Fragments of amyloid and tau may in turn drive hyperplasticity through enhanced gene transcription^29^. Indeed, proteins increased in subtype 1 were enriched for the transcription factors REST (*P*_adjusted_ = 0.018 × 10^−13^; Fig. 1d and Supplementary Table 7) and SUZ12 (*P*_adjusted_ = 0.016 × 10^−12^), which regulate plasticity-related processes through repression of neuronal differentiation genes^30,31^. Previous studies pointed towards REST de-repression and increases of tau and plasticity-related processes in AD brain tissue^7,32^, induced pluripotent stem cell (iPSC) neurons^33,34^ and tau tangle-bearing neurons^35^. Comparing subtype 1 increased proteins with those studies, we found an overlap five of six from Lu et al.^32^, 65 of 173 from Meyer et al.^33^ and 46 of 127 from Otero-Gracia^35^ (Supplementary Table 4, columns AZ-BB). Moreover, with the higher number of proteins that we measured compared to our previous study, we found additional mechanisms that may contribute to the plasticity response observed in this subtype. For example, subtype 1 had the highest CSF levels of the lysosomal protein PLD3. High PLD3 levels have been reported in dystrophic neurites associated with ‘amyloid axonal spheroids’^36^. Such spheroids trigger axonal remodeling and local hyperactivity^36^. Dystrophic neurites also accumulate BACE1, which is associated with increased APP metabolism^37^, and may explain the elevated BACE1 and amyloid-β levels in this subtype.

**Extended Data Fig. 3:**
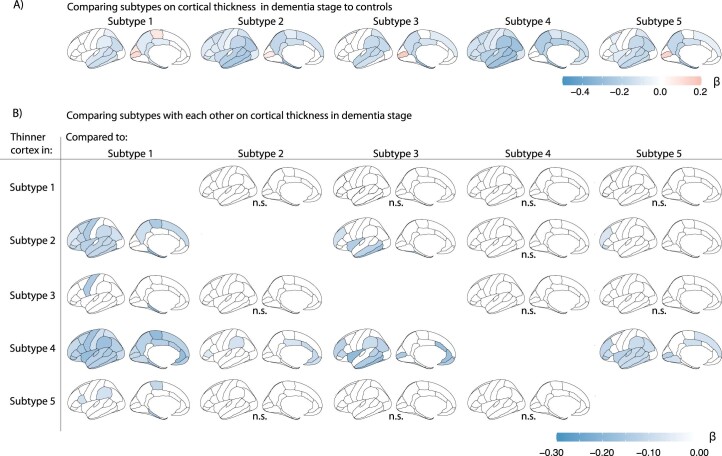
Comparing subtypes on cortical thickness in dementia stage. ß indicates mean cortical thickness in mm, averaged over right and left hemispheres and adjusted for age and sex. **a)** Cortical thickness compared between AD subtypes (in dementia stage) with controls. **b)** Cortical thickness comparisons between AD subtypes within the dementia stage. Negative values indicate thinner cortex in the subtype indicated in the row as compared to the subtype indicated in the column. Analyses were adjusted for age and sex.

**Extended Data Fig. 4:**
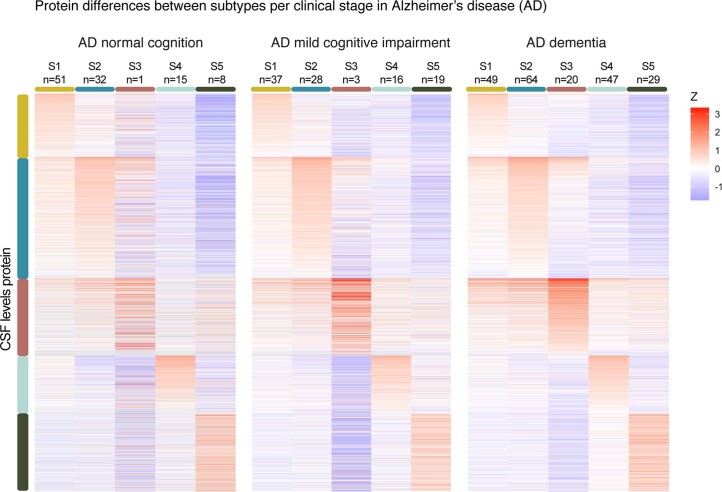
Comparing Alzheimer’s disease (AD) subtypes on protein levels against controls, plotted separately according to clinical stage. Left for cognitively normal with abnormal amyloid, middle for MCI with abnormal amyloid and right for AD dementia and abnormal amyloid. Plots indicates highly similar subtype patterns, suggesting that protein levels reflect particular AD related traits. See Supplementary Table 5 columns CY to HU for statistical metrics of subtype comparisons within each clinical stage. All proteins were scaled according to the mean and standard deviation of the control group, such that positive values indicate higher levels than controls, and negative values lower levels than controls.

Next, we tested which AD genetic risk variants^4^ were overrepresented in this subtype compared to controls. We found enrichment for *TREM2*^R47H^ and variants in *LILRB2, RHOH* and *APP* (Fig. 1f and Supplementary Table 11a). This subtype also included three of the four *PSEN1* carriers and three of the four *NCK2* carriers (Supplementary Table 11b). TREM2 is a transmembrane protein that can activate microglia when ligands, including amyloid fibrils, bind to its extracellular part^14^. The R47H variant alters the extracellular part, decreasing its ability to bind ligands, resulting in dampened microglia activation^38,39^. *LILRB2* mediates TREM2 signaling and has also been associated with dampened immune activation^40,41^. *RHOH* and *NCK2* encode signaling molecules downstream from TREM2 that influence cytoskeleton rearrangement of microglia, which enables migration toward pathogens and amyloid plaques^42^. Normally, activated microglia form a tight barrier around plaques, which decreases plaque surface and minimizes plaque contact with neurites^14,38,43^. When microglial activation is dampened, as observed in carriers of *TREM2* variants, amyloid plaques are less compact, with toxic oligomers sticking out that could damage nearby neurites^44^ and may lead to axonal dystrophy^44^, possibly triggering a plasticity response as an attempt to repair. *TREM2* has also been implicated in impaired microglial synaptic pruning, which could further contribute to the hyperplasticity signature observed in this subtype^45–47^. Such an excess of synapses was previously associated with milder atrophy in *TREM2* mouse models^45^. MRI analyses in our data indicated that this subtype had less atrophy compared to the other subtypes (Extended Data Fig. 3 and Supplementary Table 9), and was restricted to the temporal and parietal lobes.

Together, our results provide further support for a hyperplasticity subtype in AD, and provide additional insights into the underlying mechanisms, such as that this subtype could be related to a dampened microglial response. Therapies boosting TREM2 activation are under development^48^. We argue that individuals with this subtype may also respond to such treatments, even without carrying the *TREM2* R47H variant.

### Subtype 2 innate immune activation

Subtype 2 individuals (*n* = 124, 29.6%) had, compared to controls, 986 proteins with increased CSF levels and 506 with decreased levels. A high proportion of proteins increased in subtype 2 was specific to microglia. Proteins with increased levels were associated with innate immune activation, including regulation of cytokine production. These included proteins from the complement complex (C1QA, C1QB, C1QC, C1S and C1R), as well as APOE and LPL, in line with our previous findings. Additionally, we now observed that this subtype also had increased levels of the microglial Tyro3, Axl and Mer (TAM) receptors AXL and MERTK, and GAS6 (a MERTK ligand), which can detect and engulf plaques^49^. We further found increased PYCARD levels specifically in subtype 2. PYCARD is also known as apoptosis-associated speck-like protein containing a CARD (ASC), and is released by microglia with NLRP3 inflammasome activation^50,51^. PYCARD can form ASC specks, which are fibrils that worsen amyloid aggregation^51^ and induce tau phosphorylation^52^, providing a potential mechanism through which microglial activation may aggravate AD pathology. Indeed, subtype 2 individuals had higher p-tau levels than seen in subtype 1 (Table 1). Other subtype 2 increased proteins were related to neuron-microglia signaling, including CSF1, CSF1R and CX3CL1. Neuroimmune signaling occurs during normal neuronal development when microglia prune immature synapses^53–55^. In AD, activated microglia near diffuse and neuritic plaques may lead to excessive synaptic pruning^53^. This could lead to exacerbated atrophy as shown in mouse models^56^. In line with those models, subtype 2 was one of the two subtypes with the most severe and widespread cortical atrophy on MRI compared to subtypes 1, 3 and 5 (Extended Data Fig. 2). Still, despite this severe atrophy, the levels of proteins related to neuroplasticity were increased in this subtype and these proteins overlapped with subtype 1; they were also enriched for the transcription factors REST and SUZ12. Possibly, the increase of plasticity-related proteins may reflect an attempt to repair synaptic contacts, which succumbs in the presence of activated microglia. Alternatively, increased protein levels may reflect neuronal loss.

The AD genetic variants associated with this subtype were *IDUA, CLNK* and *SCIMP*, which are all involved in immune processes^4,57^.

Together, these results give additional detailed insights into the innate immune activation AD subtype and suggest that an overactive innate immune system worsens the disease.

### Subtype 3 RNA dysregulation

Subtype 3 (*n* = 24, 5.7%) emerged as one of the two additional subtypes. Compared to controls, this subtype had increased CSF levels for 516 proteins and decreased levels for 757 proteins. Proteins with increased levels were associated with cytoskeleton organization, axonal transport, and proteasome and protein folding (Supplementary Tables 4 and 9). This subtype had the highest t-tau and NEFL CSF levels. BACE1 levels were higher than in controls (Table 1), but unlike subtypes 1 and 2, amyloid-β_40_ levels were similar to controls, suggesting a different mechanism associated with higher BACE1 levels for this subtype. Proteins specifically increased in subtype 3 included heterogenous nuclear ribonucleoproteins (hnRNPs) and other RNA-binding proteins, which may point to RNA dysregulation. HnRNPs are involved in the maturation of pre-mRNAs, mRNA stabilization during transport and local mRNA translation for many RNAs, including those important for cytoskeleton organization^58^. Disruptions in hnRNPs and mRNA have been associated with tau tangles in previous proteomic studies^59^. Mislocalized hnRNPs could result in dysfunctional proteins due to mis-splicing or cryptic splicing^60^. For example, TDP43 mislocalization can lead to cryptic splicing of STMN2 (ref. ^61^), resulting in shorter proteins and decreased STMN2 levels in tissue^62^. STMN2 was detected in a subset (*n* = 84) of our sample, and subtype 3 had decreased levels of STMN2 compared to controls. Transcription factors associated with subtype 3 increased proteins were KLF4 (*P*_adjusted_ = 0.02 × 10^−15^), which is associated with axon regeneration^63^, and TAF1 (*P*_adjusted_ = 0.008 × 10^−13^) and MYC (*P*_adjusted_ = 0.02 × 10^−10^), which are interacting factors in cell differentiation processes^64,65^. A previous gene expression study in brain tissue found a similar AD subtype with increased TAF1 and MYC signaling and aberrant synapse organization^7^.

When testing AD genetic risk factors, we found enrichment for *BIN1*, which is known as ‘Myc-box-dependent interaction protein’. One of BIN1’s functions is to physically inhibit MYC^66^. BIN1 mainly localizes in axons and has many isoforms arising from splicing^66^. BIN1 mis-splicing has been associated with de-inhibition of MYC and cytoskeleton disruption^67^. *TREM2* R62H was also associated with this subtype. Other genetic risk variants associated with subtype 3 included *SPDYE3*, involved in the cell cycle, *SNX1*, important for endosome sorting, and *KAT8,* a lysine acetyltransferase^4,57^.

While RNA dysfunction has been mainly observed in frontotemporal dementia^68^, these processes have also been observed in AD in tissue^5^ and tau tangle proteomic studies^59^; our results suggests that this can be detected in the CSF of a specific AD subtype.

### Subtype 4 choroid plexus dysfunction

Subtype 4 (*n* = 78, 18.6%) was the other additional subtype. Compared to controls, this subtype had increased CSF levels of 467 proteins and decreased levels of 626 proteins. A high proportion of proteins increased in subtype 4 were specific to microglia and other immune cells. Moreover, a large subset of proteins with increased levels (45%) was associated with high expression in the lateral ventricle choroid plexus (Supplementary Table 4), including TTR, SPARC and extracellular matrix proteins such as DCN, LUM and COLA12. Biological processes associated with subtype 4 included cell adhesion, and BMP and SMAD pathways, which are involved in choroid plexus development^69^. The choroid plexus is located along the ventricles, where it produces CSF and is responsible for nutrient, lipid and protein transfer across the blood–CSF barrier^69^. It consists of a highly developed extracellular matrix that connects a dense vasculature to its epithelial cells^69^. On MRI, subtype 4 had the largest choroid plexus volume (Fig. 2b). Increased choroid plexus volume has been associated with inflammation and structural alterations in AD^70,71^. Although this subtype most often had normal t-tau and p-tau levels (Table 1), it had worse atrophy than subtypes 1, 3 and 5, with specific involvement of anterior cingulate areas (Extended Data Fig. 3). Furthermore, proteins increased in subtype 4 were enriched for fibroblasts (Supplementary Table 4), which produce extracellular matrix proteins and provide structural support to the choroid plexus. Other proteins increased in subtype 4 included cytokines, such as CCL2, CCL21 and CCL15, which can attract monocytes and T lymphocytes^72^. Of note, proteins with decreased levels in subtype 4 were related to axonal outgrowth and synaptic plasticity (for example, BDNF), in part overlapping with proteins increased in subtypes 1 and 2, and were also enriched for REST and SUZ12. This suggests that subtype 2 is also characterized by neuronal hypoplasticity.

When testing AD genetic risk variants, we found enrichment for *ABCA7*, *PICALM*, *IL-34* and *CLNK*. While *ABCA7* and *IL-34* are expressed in the choroid plexus^73,74^, *PICALM* is expressed in the blood–brain barrier^75^. Both *ABCA7* and *PICALM* have a role in lipid metabolism^76,77^ and have been associated with amyloid clearance in combination with LRP1 (ref. ^78^). *IL-34* has been associated with impaired macrophage function^79^, which could interfere with the macrophage uptake of amyloid fibrils^80^. Together with the decreased levels of BACE1 and amyloid-β_40_ in this subtype compared to controls (Table 1), suggesting decreased amyloid metabolism, these genetic factors suggests that impaired clearance mechanisms contribute to AD pathogenesis in subtype 4.

Taken together, these results suggest that choroid plexus dysfunction is another contributor to AD, in a specific subgroup of patients.

### Subtype 5 blood–brain barrier dysfunction

Subtype 5 (*n* = 56, 13.4%) was highly similar to our previously identified blood–brain barrier dysfunction subtype with increased levels of 640 proteins that included blood proteins such as albumin, fibrinogens, plasminogen, prothrombin and many immunoglobulins, such as IgG1, all proteins that leak into the brain when the blood–brain barrier is compromised^81^. Pathways associated with increased proteins included blood coagulation, B cell-mediated immunity and acute inflammatory response. No transcription factor enrichment was observed for proteins with increased CSF levels. On MRI, this subtype had more microbleeds than controls (*P* = 0.01; Table 1), unlike the other subtypes. Most proteins associated with subtype 5 (1,013, 61%) had, however, decreased CSF levels compared to controls, and these were associated with neuroplasticity and converged on the transcription factors SUZ12 and REST. This suggests that the blood–brain barrier subtype also has hypoplasticity, like the choroid plexus subtype. Neuronal plasticity processes can be impaired by leakage of blood proteins, including fibrin, which were specifically increased in this subtype^82^. Furthermore, compared to our previous study, we found additional proteins altered in the blood–brain barrier subtype, which were associated with pericytes, cells that normally cover capillaries, and with particular vascular cell types, such as lower levels of PDGFRB, CDH2 (N-cadherin), MFGE8 (medin), HTRA1, LAMB1 (laminin), EDN1, LRP1 and JAM3, as well as increased levels of CDH5 (VE-cadherin), ANXA3, ICAM1, AMBP, VWF and PTPRB (Supplementary Table 4, columns AV-AX). All of these have been previously associated with deposition of blood proteins in the parenchyma^75,83–87^. The low PDGFRb levels we observed may reflect loss of pericytes, which is in line with brain tissue measures of PDGFRb in rodent models and postmortem AD^83,88^. A decreased number of pericytes might also explain the decreased levels of LRP1 we observed in this subtype, which may impede amyloid clearance across the blood–brain barrier^78^. Alternatively, the low concentrations we observed in the blood–brain barrier subtype could reflect loss of other vascular cells, such as arterial smooth muscle cells, which also express PDGFRb^86^.

In terms of genetic risk, this subtype had the highest proportion of *APOE* e4 carriers, although the difference with other subtypes did not reach statistical significance (Table 1). This subtype was further enriched for the *IL-34*, *ECHDC3* and *APP* variants*. IL-34* was also associated with the choroid plexus subtype, suggesting that it contributes to AD pathogenesis through processes related to the brain barrier. *ECHDC3* has been associated with lipid metabolism^57^. Some variants in *APP* have been associated with vascular disruption or increased occurrence of cerebral amyloid angiopathy, through amyloid fragments that are more difficult to clear^89^. The notion that this subtype has blood–brain barrier dysfunction, suggests that this common *APP* variant may contribute to AD risk through vascular integrity.

Together, these data provide additional insights into the underlying pathophysiological processes associated with the blood–brain barrier dysfunction AD subtype.

### Predicting AD subtypes in replication cohorts

**Fig. 3:**
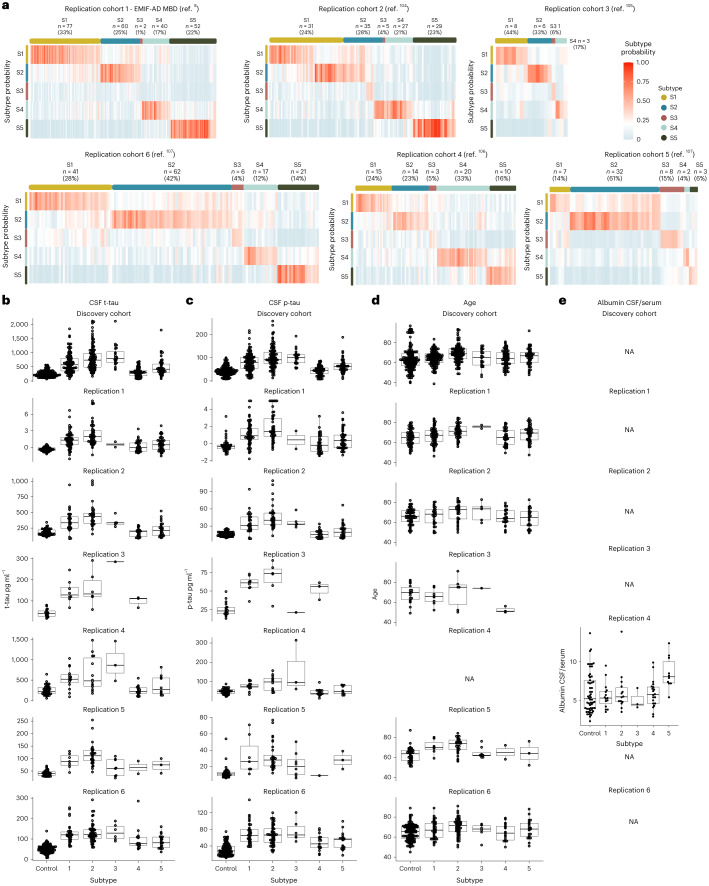
Replication of AD subtypes in six cohorts. **a**, Subtype probability for each individual in six replication cohorts. Most individuals showed high probability for one subtype only. **b**–**e**, Subtype comparisons on CSF t-tau levels (**b**), p-tau (**c**), age (**d**) and the CSF over serum albumin ratio (**e**) for each replication cohort when available. **b**–**e**, The boxplots depict the median in the center, the boundaries indicate the first and third quartiles, the whiskers extend up and down to 1.5 times the interquartile range (limited to actual observed data points) and the points indicate individual person values. The number of individuals per group in the boxplots are listed in Supplementary Tables 12 and 13, and provides the statistical metrics for the comparisons. NA, not applicable.

We then studied if the subtypes could be identified in six independent replication datasets with available CSF TMT MS. To this end, we trained random forest classifiers on the current dataset and then used these classifiers to predict subtype labels for individuals in the replication cohorts, including patients from over nine different countries in Europe and the USA (Methods). All five subtypes were observed in most replication cohorts with high subtype-specific probabilities and comparable frequencies as in the discovery cohort: on average 27.9% had subtype 1, 35.5% subtype 2, 5.8% subtype 3, 17.1% subtype 4 and 16.6% subtype 5 (Fig. 3a and Supplementary Table 12). Subtype comparisons on CSF t-tau and p-tau levels, as well as age and sex, indicated mostly similar differences as observed in the main analyses for the replication cohorts (Fig. 3b–d and Supplementary Table 13). Replication cohort 4 also had CSF over the serum albumin ratios available (Fig. 3e), which is a marker for blood–brain barrier leakage; this was increased in the blood–brain barrier subtype only (*P* < 0.0001). Overall, these results suggests that the subtypes we discovered in the main analyses are also present in other cohorts.

## Discussion

In this study, we dissected AD disease heterogeneity in patients with CSF proteomics, at a level of detail that approaches the level of complexity achieved in tissue proteomics^2,6^. Our analyses included more proteins and individuals than in our previous study and this led to a more in-depth characterization of three previously identified CSF subtypes (that is, hyperplasticity, innate immune activation and blood–brain barrier dysfunction); two additional subtypes emerged, one with RNA dysregulation, which showed the most aggressive disease course, and one with choroid plexus dysfunction. Notably, we found that each subtype was associated with distinct AD genetic risk factors, further supporting that each CSF AD subtype reflects specific underlying molecular mechanisms. The subtypes also differed in cortical atrophy patterns and survival times, underscoring their clinical relevance.

A potential limitation of our study is that AD was defined with biomarkers, which correlate strongly with pathology but sometimes may be inaccurate. Another limitation is that we were unable to test if subtypes would have a different treatment response because we did not have access to samples from trials. Given the distinct patterns of molecular processes and AD genetic risk profiles, it is likely that AD subtypes will require specific treatments. For example, subtype 1 individuals may benefit from TREM2-activating treatments, subtype 2 from innate immune inhibitors, subtype 3 from antisense oligonucleotides that restore RNA processing, subtype 4 from inhibition of monocyte infiltration and subtype 5 from cerebrovascular treatments. At the same time, side effects arising from certain treatments may also depend on subtype. For example, while antibodies may more easily cross the blood–brain barrier in subtype 5, these individuals may be at increased risk for cerebral bleeding that can occur with antibody treatment. Future studies should aim to (re)analyze proteomics in clinical trial samples to test whether particular treatments have subtype-specific effects. To conclude, CSF-based subtyping may be useful to select individuals for a specific therapeutic treatment, either for a priori subject stratification or for responder and side effect analysis in clinical trials.

## Methods

### Participants

For this study, we selected individuals from studies performed at the Alzheimer Center Amsterdam under similar protocols (that is, Amsterdam Dementia Cohort (ADC)^15^, EMIF-AD preclinAD^16^ and 90+ studies^17^, and Amsterdam site participants who coenrolled in the ADC biobank and the EPAD study^18^) who provided written informed consent to use their data and biospecimens for research purposes. None of the individuals overlapped with our previous CSF proteomics study^8^. AD was defined based on the presence of an abnormal amyloid marker (*n* = 419), and controls were required to have normal cognition with normal amyloid markers (described in the ‘CSF ELISA measures’ section). Among the group with abnormal amyloid markers, 107 individuals had normal cognition, 103 had MCI and 209 had dementia according to international consensus criteria^15–18^. When more individuals met criteria for inclusion, preference was given to individuals with a known *TREM2* R47H (*n* = 8) or R62H mutation (*n* = 28; see the details in the ‘Genetic data’ section), to individuals without dementia with clinical follow-up (*n* = 216) and to individuals with available tau positron emission tomography (PET) (*n* = 36). Mortality information was obtained from the Dutch Municipal Register for ADC and EMIF-AD participants. All studies were approved by the ethics committee of the Amsterdam UMC (location VUmc), the Biobank Research Ethics Committee of the Amsterdam UMC (location VUmc) and the Ethics Committee of the University of Norway.

### CSF collection and preparation for MS

CSF was collected by lumber puncture between the L3/L4, L4/L5 or L5/S1 intervertebral space with a 25-gauge needle and syringe and collected in polypropylene tubes^15^. For all participants, CSF sample processing and biobank storage at the Alzheimer center biobank at the department of Laboratory Medicine was performed according to international guidelines^15^. The 610 samples were randomized over seven 96-well plates using random sampling as implemented in R to determine the layouts, 100 µl CSF in each well and stored at −80 °C. Researchers measuring the CSF samples were blinded to the diagnosis. Each sample plate was thawed on ice and 30 µg protein (separate 96-well plates containing 40 µl CSF were used to carry out the bicinchoninic acid (BCA) protein assay on 2 × 10 µl CSF for the protein concentration measurements) from each well was transferred to 1.5 ml protein LoBind tubes and immediately frozen on dry ice. All samples were lyophilized using a freeze dryer and kept at −80 °C before digestion. Urea protein digestion was performed as follows. Each day until there were no more samples to process, 28 samples together with one quality control sample and two reference samples were added (20 µl of 8 M urea/20 mM methylamine), vortexed for 5 min at 1,000 r.p.m. and sonicated for 30 s in ice-cold water. The urea solution was diluted with 20 µl of 50 mM Tris HCl/1 mM CaCl_2_, pH 7.6, followed by cysteine reduction (0.4 µmol dithiothreitol (DTT), 1 h incubation at room temperature) and alkylation (1 µmol iodoacetamide, 1 h incubation in the dark at room temperature). To avoid protease alkylation, excess iodoacetamide was allowed to react with DTT by adding 0.08 µmol of the reagent before diluting the urea to 1 M with 50 mM Tris HCl/1 mM CaCl_2_, pH 7.6. Trypsin digestion was performed for 16 h at 37 °C after adding 0.6 mg of the protease (porcine trypsin, Promega Corporation). Sample cleanup was performed using a reverse-phase Oasis 96-well HLB μElution Plate 30 μm (2 mg HLB sorbent, Waters). After lyophilization, the quality control samples were resuspended in 25 µl 2% acetonitrile (ACN)/0.5% formic acid. All other samples were resuspended in 20 µl of 50 mM HEPES buffer, pH 8.2, 4-(2-hydroxyethyl) 2-piperazin-1-ylethanesulfonic acid) before TMT labeling. Samples were vortexed for 30 s at 1,500 r.p.m. and sonicated for 30 s in an ultrasonic bath. Each reporter in a 5-mg TMTpro 16plex reagent set was dissolved in 1 ml anhydrous ACN. The 610 samples were labeled in 44 experiments, where each experiment contained 14 samples and 2 reference samples. For each sample, the 20-µl label was added. The labeling reaction was stopped by adding 5 µl 5% hydroxylamine after 75 min. The 16 labeled samples for each experiment were combined and lyophilized (about 240 µg protein); approximately 150 µg were desalted using a reverse-phase Oasis 96-well HLB Elution Plate 30 μm (10 mg HLB sorbent, Waters). After lyophilization, the 44 samples were dissolved in 150 µl of 10 mM ammonium formate, pH 7.9; 65 µl were fractionated using an off-line high-performance liquid chromatography (HPLC) system (Agilent 1260 infinity, Agilent Technologies) equipped with a reverse-phase column (XSelect CSH C18, 130 Å, 3.5 µm, 1 × 150 mm, Waters). Using high-pH reverse-phase chromatography, peptides were separated during a biphasic ACN gradient from two HPLC pumps (flow rate of 50 µl min^−1^). Solvents A and B were 10 mM ammonium formate, pH 7.9, in water and 90% ACN/10% water, respectively. The gradient composition was 5% B during trapping (2 min) followed by 5–12% B over 1 min, 12–44% B for the next 35 min, 44–70% B over 10 min and 70–95% B over 2 min. Elution of very hydrophobic peptides and conditioning of the column were performed for a 5-min isocratic elution with 95% B and a 12-min isocratic elution with 5% B, respectively. Peptides were collected in a 500-µl protein LoBind 96-well plate during peptide elution; ten fractions were collected. The first fraction was collected in two wells from 5 to 16 min (5.5 min per well, merged into one fraction); the next eight fractions (2.7 min per fraction) were collected between 16 and 37.6 min; the last fraction was collected in two wells between 37.6 and 53.6 min (8 min per well, merged into one fraction). Fractions were lyophilized and resuspended in 10 µl 2% ACN/0.5% formic acid, and peptide concentrations were measured with a NanoDrop UV-Vis spectrophotometer (Thermo Fisher Scientific) before LC–MS/MS analysis.

### LC–MS/MS

About 0.5 μg tryptic TMT-labeled peptides were injected into an Ultimate 3000 RSLC system (Thermo Fisher Scientific) connected online to a Exploris 480 mass spectrometer (Thermo Fisher Scientific) equipped with an EASY-Spray nano-electrospray ion source. Peptides were desalted on a precolumn (Acclaim PepMap 100, 2 cm × 75 µm ID nanoViper column, packed with 3 µm C18 beads) at a flow rate of 5 µl min^−1^ for 5 min with 0.1% trifluoroacetic acid before separation on a 50-cm analytical column (PepMap RSLC, 50 cm × 75 µm ID EASY-Spray column, packed with 2 µm C18 beads). During a biphasic ACN gradient from two nanoflow UPLC pumps (solvent A and B were 0.1% formic acid (vol/vol) in water and 100% ACN, respectively), peptides were separated through the reverse-phase column at a flow rate of 200 nl min^−1^. The gradient composition was 5% B during trapping (5 min) followed by 5–8% B over 1 min, 8–30% B for the next 104 min, 30–40% B over 15 min and 40–80% B over 3 min. Elution of very hydrophobic peptides and conditioning of the column was performed for a 9-min isocratic elution with 80% B and a 10-min isocratic elution with 5% B. The LC was controlled through an SII for Xcalibur 1.6 (Thermo Fisher Scientific).

Peptides eluted from the column were detected in the Exploris 480 mass spectrometer (capillary temperature at 275 °C and ion spray voltage at 2100 V) with high-field asymmetric waveform ion mobility spectrometry (FAIMS) enabled using two compensation voltages (CVs) (−50V and −70V), and ‘advanced peak determination’ on. During each CV, the mass spectrometer was operated in the data-dependent acquisition (DDA) mode to automatically switch between one full scan MS and MS/MS acquisition. Instrument control was through an Orbitrap Exploris 480 Tune 3.1 and Xcalibur 4.4. The cycle time was maintained at 1.5 s per CV. The FAIMS filter performed gas-phase fractionation, enabling the preferred accumulation of multiply charged ions to maximize the efficiency of the DDA. FAIMS results in less precursor coisolation and cleaner MS/MS spectra. MS spectra were acquired in the scan range of 375–1,500 *m*/*z* with resolution *R* = 60,000 at 200 *m*/*z*, automatic gain control target of 3 × 10^6^ and a maximum injection time at auto (depending on the transient length in the Orbitrap). The most intense eluting peptides with charge states 2–6 and above an intensity threshold of 2 × 10^4^ were sequentially isolated to a standard target value of 2 × 10^5^, or a maximum injection time of 120 ms in the C-trap, and isolation width maintained at 0.7 *m*/*z* (quadrupole isolation), before fragmentation in the higher-energy collision dissociation. Fragmentation was performed with a normalized collision energy of 32%; fragments were detected in the Orbitrap at a resolution of 45,000 at 200 *m*/*z*, with first mass fixed at 110 *m*/*z*. One MS/MS spectrum of a precursor mass was allowed before dynamic exclusion for 45 s with ‘exclude isotopes’ on. Lock-mass internal calibration was not enabled.

The resulting .raw files were processed using Proteome Discoverer 2.5. The database file used for the search using Sequest HT was Swiss-Prot with 20,395 entries (v.20210413.fasta). The following modifications were made in the database search: precursor mass tolerance: 10 ppm; fragment mass tolerance: 0.02 Da; static peptide N terminus: TMTpro/ + 304.207 Da (any N terminus); static modification: TMTpro/ + 304.207 Da (K); static modification: carbamidomethyl (C); and dynamic modification for methionine oxidation. The maximum of missed cleavage cites was set to 2, with a minimum peptide length of 6. The validation settings were set to 0.01 for strict PSM false discovery rate (FDR) using a target-decoy strategy and 0.05 for relaxed. The peptides used were set to unique + razor. Reporter abundance was based on intensity with a coisolation threshold of 50 and average reporter S/N threshold of ten. The output files where then gathered and subjected to further processing.

### CSF enzyme-linked immunosorbent assay

Amyloid-β_42_, t-tau and p-tau were previously determined using an enzyme-linked immunosorbent assay (ELISA) from Innotest (Fujirebio, formerly Innogenetics) in the ADC, or with the Roche Elecsys System (*n* = 15 from ADC and in EPAD). In the EMIF-AD preclinAD study, amyloid status was determined based on the amyloid-β_42_/amyloid-β_40_ ratio, which, together with t-tau and p-tau 181, were measured with ELISAs from ADx NeuroSciences/EUROIMMUN. In the EMIF-AD 90+, amyloid status was determined with visual reading of flutemetamol (^18^F) PET. For these individuals (*n* = 22), tau levels were computed from the TMT microtubule-associated protein tau (MAPT) measures, which correlated strongly (*r* = 0.81, *P* < 0.001) with the Innotest t-tau levels in the ADC cohort (formula: Inno t-tau = −309.16 + 0.01 × MAPT). For tau categorization, we used t-tau values because these were available for all individuals and correlated strongly with p-tau levels (*r* = 0.94, *P* < 0.001). We used published cutoffs to label individuals as having a normal AD CSF profile or abnormal amyloid based on CSF^90–94^. Three individuals with normal cognition had normal amyloid and abnormal CSF t-tau levels, which were excluded from the present analyses, resulting in a final sample size of 187 controls and 419 individuals with abnormal amyloid. We standardized continuous amyloid 1–42, t-tau and p-tau 181 values within specific assays according to the mean and s.d. of controls to compare these values between subtypes. Finally, we measured BACE1, amyloid-β_40_ and neurogranin with EUROIMMUN ELISA assays (Germany), NEFL with ADx NeuroSciences (Belgium) ELISA assay and VAMP2 with a prototype assay developed by ADx NeuroSciences (Belgium) with single-molecule array technology (Quanterix Corporation) as described previously^95^. These measures were not included in clustering, but used as independent markers to compare AD subtypes.

### Genetic data

*APOE* genotyping was performed in blood. A subset of 560 individuals had genotyping data available (Illumina Global Screening Array). Details on quality control procedures were described previously^96^; for EPAD, they are available on GitHub at https://github.com/marioni-group/epad-gwas). Genotype VCF files were imputed using the TopMed reference panel. Eighty-three genetic risk loci for AD were selected based on their previous genome-wide association with AD^4^. These single-nucleotide polymorphisms (SNPs) were extracted from the genetic data based on rsID or base pair location in the genome. Protective SNPs (that is, odds ratios < 1) were inverted, such that for all SNPs higher values indicate higher AD risk.

### MRI data

A subset 503 individuals had structural T1-weighted MRI available. To test if subtypes were characterized by different atrophy patterns, we restricted analyses to subtypes in the dementia stage (*n* = 159 and 160 controls) because in that stage atrophy is most pronounced. Acquisition details were described previously^16,97–99^. Cortical thickness, hippocampal volume, choroid plexus volume and total intracranial volumes were estimated with FreeSurfer v.7.1.1 (http://surfer.nmr.mgh.harvard.edu/). Cortical thickness and volumetric estimates were summarized in anatomical regions as defined by the FreeSurfer implementation of the Desikan–Killiany atlas. Choroid plexus and hippocampal volumes were adjusted for total intracranial volume to adjust interindividual differences in head size. Furthermore, microbleeds were counted on T2* sequences by an experienced neuroradiologist and defined as small round hypointense foci up to 10 mm in the brain parenchyma.

### Longitudinal cognitive assessment

Most individuals had clinical follow-up available that was planned approximately on a yearly basis. Follow-up diagnoses were based on the same criteria as described above for the baseline diagnosis. Cognitive functioning was assessed globally with the MMSE and for specific cognitive domains with standardized neuropsychological test batteries at the first visit and repeated over time^15–18^. We selected tests that were comparable across substudies: for memory, the immediate and delayed recall of the Dutch version of the Rey auditory verbal learning tasks (ADC and EMIF-AD preclinAD), the Consortium to Establish a Registry for Alzheimer’s Disease 10-word test (EMIF-AD 90+) or the RBANS 10-word list learning (EPAD); for language, the 1-min animal fluency test (ADC, EMIF-AD preclinAD and 90+) and the Repeatable Battery for the Assessment of Neuropsychological Status 1-min category fluency (EPAD); for attention, the TMT-A test (ADC, EMIF-AD preclinAD and 90+ only); and for executive functioning, the TMT-B test (ADC, EMIF-AD preclinAD and 90+ only). The TMT-A and TMT-B scores were inverted so that, like the other tests, lower scores indicate worse performance. All neuropsychological test scores were standardized according to the mean and s.d. of the baseline scores of the control group in each substudy separately. Baseline animal fluency scores were missing for EMIF-AD preclinAD individuals, which were imputed before standardizing.

### Statistics and reproducibility

**Extended Data Fig. 5:**
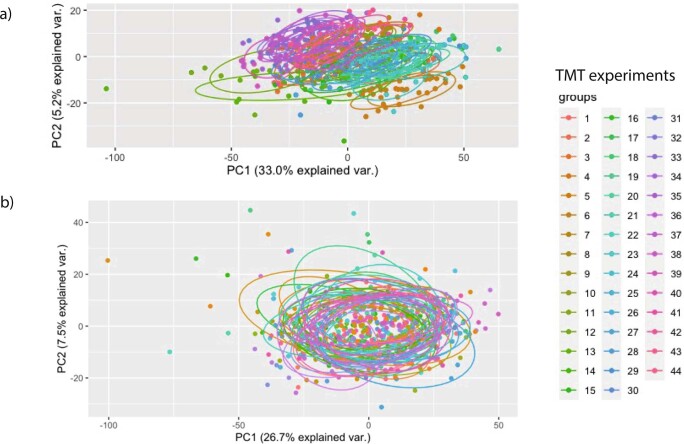
Batch correction of TMT experiments. **a**) Biplot of first two principal components on unnormalized protein abundances have batch effects between TMT experiments as indicated by the non-overlapping circles that correspond to all 44 TMT experiments. **b**) Batch effects were successfully removed with the Internal Reference Scaling method as described in the online methods.

Sample size for the present analyses was determined before this study, using a simulation study based on our previous results, which included 284 individuals with three subtypes^8^. Simulating a protein correlation structure with 4–6 additional subtypes required 300–400 participants. Therefore, we selected 419 individuals with AD and 187 controls. The researchers measuring the proteomics in CSF were blinded to the diagnosis. The researchers performing the statistical analyses were not blinded to the diagnosis because the diagnosis was required for the subgroup analyses. All samples were randomized over the TMT experiments using random sampling as implemented in R to determine the layouts. One person for whom proteomics was measured (selected based on the presence of tau PET) was excluded from further analyses in this study because they had normal AD markers in CSF but a clinical diagnosis of primary progressive aphasia, leaving 609 individuals included for the statistical analyses. Because we performed data-driven analyses to study clusters in the data, no randomization method was used to allocate individuals to the experimental groups. Technical deviations may have influenced protein abundance across the TMT experiments. Before the statistical analyses, we normalized protein abundance according to the internal reference scaling normalization procedure^100^ for TMT proteomics data that use the common pool reference channels to normalize values between plex experiments, adapted to scale according to the median instead of the total sum to reduce the influence of outliers. Briefly, the first step in this two-step approach normalized the grand total protein intensities for each of the 14 channels within an experiment to match these to the two reference channels. In the second step, a correction factor was calculated based on common pooled internal standards to normalize reporter ion intensities of proteins between TMT experiments. Internal standards were unavailable for 113 proteins, which were excluded from the subsequent analyses. Extended Data Fig. 5 illustrates that batch effects were removed. Next, protein values were log_2_-transformed and then scaled according to the mean and s.d. of the control group, so that positive and negative values indicate higher and lower than normal. For all proteins, we report gene names to aid comparisons with other AD subtyping literature using either proteomics or RNA-seq data.

### AD subtype discovery

Our objective was to identify subtypes within AD, and so we first selected proteins that were related to AD. For this, we compared all AD individuals to controls with regard to the CSF levels of proteins that were observed in the complete sample with Kruskal–Wallis tests. Because previous studies indicated that AD-related alterations of CSF protein levels may depend on cognitive state or tau status in a nonlinear way^12^, we repeated these analyses stratified for these factors. This resulted in 1,058 proteins that were selected for clustering with nonnegative matrix factorization as implemented in the NMF package v.0.25 in R v.4.2.2. Bird Hippie. We followed the procedure as in our previous study^8^. Briefly, proteins were first scaled to have positive values between 1 and 2, keeping the relative values intact. Next, we performed 30 different runs of NMF to determine the number of clusters (that is, the subtypes) that best described the data. We tested up to ten clusters, and found that five clusters showed an optimal balance of a high cophonetic coefficient, at least twofold improved fit over the lower clustering solution, compared to improvement using a random cluster solution, and a silhouette score greater than 0.5 (Supplementary Table 3). To test the robustness of clusters, we repeated clustering using the Louvain algorithm on the protein coexpression networks, excluding the diagonal and setting the resolution parameter to 1.15, as implemented in the igraph R package v.1.3.2. Next, we labeled each individual patient according to the subtype that best matched their proteomics profile. Patient-level subtype clusters were visualized by projecting the NMF subtype scores to a UMAP embedding, via construction of a *k*-nearest neighbor graph using the uwot R package v.0.1.14. Patient-level subtype labels provide the basis for all subsequent post hoc comparisons described in the next sections.

### Biological characterization of AD subtypes

We characterized the biological processes associated with AD subtypes by comparing the subtypes on CSF protein levels of all available proteins, including, in addition to the fully observed proteins, also proteins with missing values when they had at least five observations available in each subtype group (2,907 proteins in total). For this we used linear models with participant subtype status as predictors and protein levels as outcomes. We repeated the analyses correcting for age and sex, and stratifying according to cognitive state to determine the influence of these factors on the results. All subtypes were compared to the control group, as well as to each other; results from all comparisons are reported in Supplementary Table 4. We performed pathway enrichment analyses for biological processes from the GO 13 January 2022 release as accessed using Panther v.16.0, for the proteins that were associated with each subtype (that is, differed from the controls with *P* < 0.05), separately for increased and decreased alterations. A hypergeometric Fisher exact test was used for pathway enrichment and pathway *P* values were corrected for multiple testing with the FDR procedure. We further tested if AD subtype-related proteins were associated with potential upstream transcription factors from the CHEA and ENCODE databases through ENRICHR. We further annotated proteins according to cell type specificity using the Human Protein Atlas (https://www.proteinatlas.org) and the RNA-seq Barres database^101^; for specific vascular cell types with Garcia et al.^86^ and Yang et al.^75^; for choroid plexus associations according to the harmonizome database^102^; for REST signaling associations based on Meyer et al.^33^ and Otero-Garcia et al.^103^; for blood–brain barrier dysfunction according to Dayon et al.^81^; and for the CSF pathway panels informed by tissue proteomics to Higginbotham et al.^3^.

### Post hoc comparisons between subtypes on clinical characteristics

We performed post hoc tests to characterize AD subtypes clinically and biologically with chi-squared tests^2^ for discrete variables (sex and APOE e4 genotype), and with linear regression models for continuous variables correcting for age and sex when applicable. Subtype differences with regard to time to progress to dementia was tested with Cox proportional-hazards models and restricted to the prodromal stage of individuals for reasons of statistical power. Subtype differences in survival times were also tested with Cox proportional-hazards models, and restricted to individuals in the dementia stage for reasons of statistical power. Subtype differences on baseline cognitive test scores, as well as changes over time, were tested with linear mixed models, stratified according to disease stage. Subtype differences with controls on genetic variants as continuous outcomes were tested with linear regression models, taking imputation uncertainty into account when possible. Missing values (*n*) were recorded for years of education (*n* = 6), APOE genotype (*n* = 26), AD PRS (*n* = 68), CSF p-tau (*n* = 26), CSF NEFL (*n* = 3) and microbleeds on MRI (*n* = 163). All analyses were repeated with age and sex as covariates (and level of education for cognitive data).

### Predicting AD subtypes in replication cohorts

We trained random forest classifiers in the discovery cohort to predict AD subtypes in the replication cohorts that had proteomics data available with TMT MS in CSF in individuals with AD and controls. Procedures for the replication cohort 1 were previously described by Tijms et al.^8^, replication cohort 2 by Modeste et al.^104^, replication cohort 3 by Dammer et al.^105^ and Higginbotham et al.^3^, replication cohort 4 by Dayon et al.^106^ (provided with ref. ^3^), and replication cohorts 5 and 6 by Johnson et al.^107^. First, for each cohort we normalized between plex experiments in the same way in the discovery cohort. Next, we scaled proteins according to the mean and s.d. of the control group (that is, normal cognition and normal CSF amyloid and tau markers). We then determined for each cohort which proteins were observed in all samples and matched these proteins to the discovery cohort based on UniProt codes. The replication cohorts different in the proteins detected; so we trained four random forest classifiers to include as much overlapping proteins as possible across cohorts, starting with the cohort with the most overlap with the current study (that is, replication cohorts 1 and 2; see columns Y-AB in Supplementary Table 4 for the proteins included). Each random forest was trained on 80% of the discovery data and tested on the left out 20% using random sampling without replacement. For each training set, subtype frequencies were balanced across classes with SCUT (scutr R package v.0.1.2), which uses the SMOTE algorithm to simulate additional cases in minority classes, and *k*-nearest neighbor clusters to undersample the majority classes. The same training and test data were used for each of the four types of random forests to compare prediction accuracies between forests. We repeated the training and testing procedure for all random forests 100 times to determine classification stability. Random forests were trained with ntree set to 1,000 and then used for prediction with the randomForest R package v.4.7-1.1. In each replication set, we assigned individuals to the subtype with the highest predicted probability. Next, we tested if subtypes had comparable differences on CSF t-tau and p-tau levels as the discovery cohort; for replication cohort 4, subtypes were also compared on the CSF serum albumin ratio as an index of blood–brain barrier dysfunction. Note that these measures were not included in the random forests. Finally, subtypes were compared according to APOE genotype, sex and age.

### Reporting summary

Further information on research design is available in the Nature Portfolio Reporting Summary linked to this article.

### Supplementary information

Supplementary Information Supplementary Fig. 1.

Reporting Summary

Supplementary Table Supplementary Tables 1–13.

## Data Availability

All mass spectrometry data generated for this study with accompanying demographical information are available through the ADDI workbench (https://fair.addi.ad-datainitiative.org/#/data/datasets/five_csf_proteomic_subtypes_in_ad; 10.58085/HR6S-2991). Other data used in this publication were accessed as described in the Methods. All other data are available from the corresponding author upon reasonable request.
