# Supplementary material for: Cerebrospinal fluid proteomics in patients with Alzheimer’s disease reveals five molecular subtypes with distinct genetic risk profiles
Source: Nat Aging. 2024 Jan 9;4(1):33–47. doi: 10.1038/s43587-023-00550-7 (PMC10798889; doi:10.1038/s43587-023-00550-7)
Supplement: Supplementary file 1 — Supplementary Fig. 1. [file 43587_2023_550_MOESM1_ESM.pdf]

nature aging

Letter

https://doi.org/10.1038/s43587-023-00550-7

Cerebrospinal fluid proteomics in patients with Alzheimer's disease reveals five molecular subtypes with distinct genetic risk profiles

In the format provided by the authors and unedited

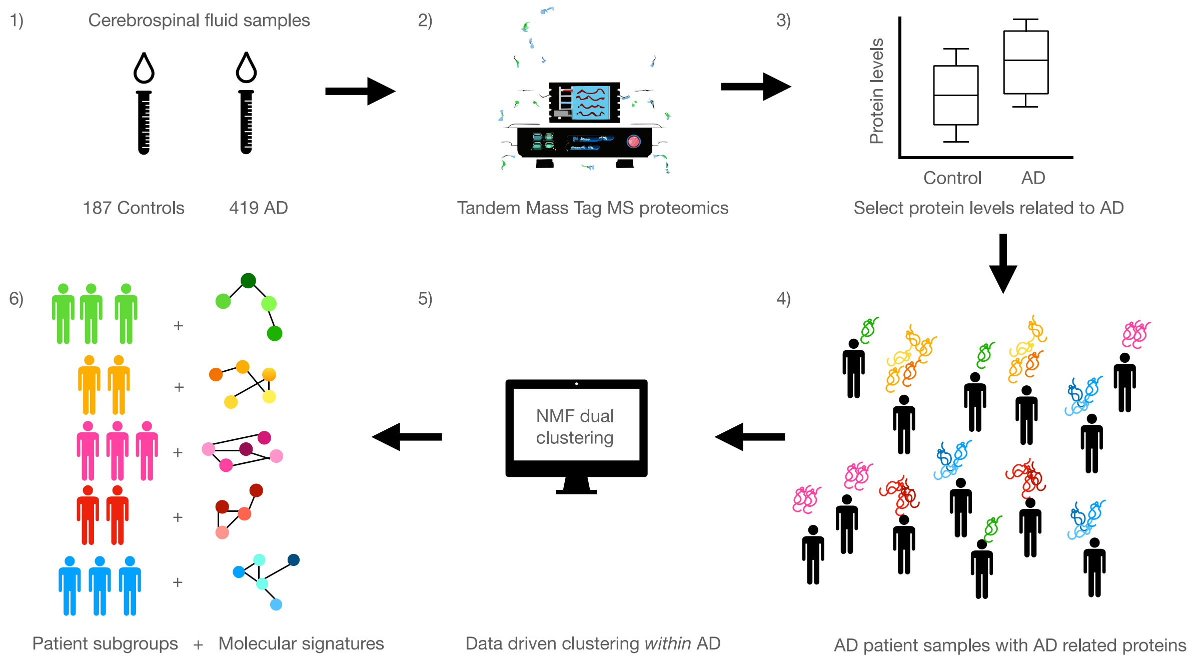

Supplementary figure 1. Schematic overview of subtype discovery within AD patients. 1) Cerebrospinal fluid (CSF) samples from 198 controls and 419 individuals with Alzheimer's disease (AD) were selected and 2) were analysed with tandem mass tag mass spectrometry to obtain untargeted proteomics. 3) Protein levels were then compared between controls and AD to select proteins associated with AD. 4) Within the AD group for proteins related to AD, data driven clustering was performed with non-negative matrix factorisation (NMF) (5). 6) The resulting patient subgroups were then molecularly characterised based on their corresponding proteomic signatures, and compared on clinical and biological characteristics.
