# Supplementary material for: Cerebrospinal fluid proteomics in patients with Alzheimer’s disease reveals five molecular subtypes with distinct genetic risk profiles
Source: Nat Aging. 2024 Jan 9;4(1):33–47. doi: 10.1038/s43587-023-00550-7 (PMC10798889; doi:10.1038/s43587-023-00550-7)
Supplement: Supplementary file 2 — Reporting Summary [file 43587_2023_550_MOESM2_ESM.pdf]

nature portfolio

Corresponding author(s): Betty Tijms

Last updated by author(s): Nov 1, 2023

Reporting Summary

Nature Portfolio wishes to improve the reproducibility of the work that we publish. This form provides structure for consistency and transparency in reporting. For further information on Nature Portfolio policies, see our Editorial Policies and the Editorial Policy Checklist.

Statistics

For all statistical analyses, confirm that the following items are present in the figure legend, table legend, main text, or Methods section.

n/a	Confirmed	
☐	☒	The exact sample size (*n*) for each experimental group/condition, given as a discrete number and unit of measurement
☐	☒	A statement on whether measurements were taken from distinct samples or whether the same sample was measured repeatedly
☐	☒	The statistical test(s) used AND whether they are one- or two-sided<br>*Only common tests should be described solely by name; describe more complex techniques in the Methods section.*
☐	☒	A description of all covariates tested
☐	☒	A description of any assumptions or corrections, such as tests of normality and adjustment for multiple comparisons
☐	☒	A full description of the statistical parameters including central tendency (e.g. means) or other basic estimates (e.g. regression coefficient) AND variation (e.g. standard deviation) or associated estimates of uncertainty (e.g. confidence intervals)
☐	☒	For null hypothesis testing, the test statistic (e.g. *F*, *t*, *r*) with confidence intervals, effect sizes, degrees of freedom and *P* value noted<br>*Give P values as exact values whenever suitable.*
☒	☐	For Bayesian analysis, information on the choice of priors and Markov chain Monte Carlo settings
☐	☒	For hierarchical and complex designs, identification of the appropriate level for tests and full reporting of outcomes
☒	☐	Estimates of effect sizes (e.g. Cohen's *d*, Pearson's *r*), indicating how they were calculated

Our web collection on statistics for biologists contains articles on many of the points above.

Software and code

Policy information about availability of computer code

Data collection	No software was used to collect data
Data analysis	Thermo Scientific SII for Xcalibur 1.6 was used to control liquid chromatography. Proteome Discoverer 2.5 was used for processing the raw MS data. Freesurfer v7.1.1 for postprocessing MRI data, R version 4.2.2 for all statistical analyses. Gene Ontology (GO) release 2022-01-13 as accessed by Panther version 16.0 was used to determine enrichment for biological pathways.

For manuscripts utilizing custom algorithms or software that are central to the research but not yet described in published literature, software must be made available to editors and reviewers. We strongly encourage code deposition in a community repository (e.g. GitHub). See the Nature Portfolio guidelines for submitting code & software for further information.

Data

Policy information about availability of data

All manuscripts must include a data availability statement. This statement should provide the following information, where applicable:

- Accession codes, unique identifiers, or web links for publicly available datasets
- A description of any restrictions on data availability
- For clinical datasets or third party data, please ensure that the statement adheres to our policy

All mass spec data generated for this study will become available through the ADDI workbench after publication of this manuscript. Other data used in this publication was accessed as described in the methods section.

Research involving human participants, their data, or biological material

Policy information about studies with human participants or human data. See also policy information about sex, gender (identity/presentation), and sexual orientation and race, ethnicity and racism.

Reporting on sex and gender	Sex was self reported. Numbers on sex are provided in table 1 and supplementary table 1.
Reporting on race, ethnicity, or other socially relevant groupings	Not applicable
Population characteristics	Relevant characteristics were reported in table 1 and supplementary table 1
Recruitment	REcruitement was reported in the methods section at p10 (and in publications cited there)
Ethics oversight	All studies were approved by the ethical Committee of the Amsterdam UMC, location VUmc, the Biobank Research Ethics Committee of the Amsterdam UMC, location VUmc, and the Ethical Committee of the University of Norway

Note that full information on the approval of the study protocol must also be provided in the manuscript.

Field-specific reporting

Please select the one below that is the best fit for your research. If you are not sure, read the appropriate sections before making your selection.

☒ Life sciences ☐ Behavioural & social sciences ☐ Ecological, evolutionary & environmental sciences

For a reference copy of the document with all sections, see nature.com/documents/nr-reporting-summary-flat.pdf

Life sciences study design

All studies must disclose on these points even when the disclosure is negative.

Sample size	Our pilot study in 284 subjects detected the presence of three AD pathophysiological subtypes (distribution 50%, 25% and 25%) that showed differences in over 100 proteins with a power of 80% and with 1-sided testing at p=0.05, suggesting that subgroups should have a minimum n=30 to discover meaningful subtype differences. Data-driven approaches require a sample size of at least n=50 to discover reproducible subtypes.[52] We performed simulation study with parameters estimated from our pilot data.[52] Increasing the sample size to n=650 and keeping the number of proteins stable resulted in discovery of an additional fourth subtype. Next we simulated the effect of the increase in number of proteins to 1000, with correlation structures to represent 5 additional subtypes (increasing the number of subtypes to 8). The sample size of 650 subjects was able detect all these additional subtypes, while in our pilot sample size (n=284) only one of these additional subtypes was detected. Together these simulation analyses suggest that our sample size of 650 substantially increases power to detect more fine-grained AD subtypes.
Data exclusions	One individual was excluded for whom proteomics was measured, but had normal CSF markers and appeared later to have a diagnosis of PPA (and so could not serve as a control)
Replication	We replicated the new subtypes in 6 independent cohorts with CSF TMT proteomics available, as described in the methods section at p16
Randomization	Samples were randomised across TMT plex channels
Blinding	Researchers who measured TMT proteomics were blinded to diagnosis. Researchers performing statistical analyses were not blinded to diagnosis, because diagnosis was required for subgroup analyses.

Reporting for specific materials, systems and methods

We require information from authors about some types of materials, experimental systems and methods used in many studies. Here, indicate whether each material, system or method listed is relevant to your study. If you are not sure if a list item applies to your research, read the appropriate section before selecting a response.

Materials & experimental systems

n/a	Involved in the study
☐	☒ Antibodies
☒	☐ Eukaryotic cell lines
☒	☐ Palaeontology and archaeology
☒	☐ Animals and other organisms
☒	☐ Clinical data
☒	☐ Dual use research of concern
☒	☐ Plants

Methods

n/a	Involved in the study
☒	☐ ChIP-seq
☒	☐ Flow cytometry
☐	☒ MRI-based neuroimaging

Antibodies

Antibodies used	Described in methods section at p 13
Validation	*Describe the validation of each primary antibody for the species and application, noting any validation statements on the manufacturer's website, relevant citations, antibody profiles in online databases, or data provided in the manuscript.*

Magnetic resonance imaging

Experimental design

Design type	*Indicate task or resting state; event-related or block design.*
Design specifications	*Specify the number of blocks, trials or experimental units per session and/or subject, and specify the length of each trial or block (if trials are blocked) and interval between trials.*
Behavioral performance measures	*State number and/or type of variables recorded (e.g. correct button press, response time) and what statistics were used to establish that the subjects were performing the task as expected (e.g. mean, range, and/or standard deviation across subjects).*

Acquisition

Imaging type(s)	Structural MRI was acquired
Field strength	1.5 or 3 T
Sequence & imaging parameters	Are detailed in the methods section at p13-14, and in the references therein.
Area of acquisition	Whole brain

Diffusion MRI ☐ Used ☐ Not used

Preprocessing

Preprocessing software	Freesurfer v7.1.1
Normalization	Volumetric data was normalised for headsize
Normalization template	DAta were compared in Desikan Killiany defined regions of interest as implemented by Freesurfer
Noise and artifact removal	n.a.
Volume censoring	n.a.

Statistical modeling & inference

Model type and settings	Detailed in the methods section at p15
Effect(s) tested	Subtypes were predictors and regions of cortical thickness or volume were outcomes

Specify type of analysis: ☐ Whole brain ☒ ROI-based ☐ Both

Anatomical location(s): Desikan Killiany atlas

Statistic type for inference

(See Eklund et al. 2016)

n.a.

Correction

These were post hoc analyses

Models & analysis

n/a	Involved in the study
☒	☐ Functional and/or effective connectivity
☐	☒ Graph analysis
☒	☐ Multivariate modeling or predictive analysis

Graph analysis

detailed in methods section at p14
